# Protocol for Developing and Validating a Multimarker-Clinical Prediction Model of SGLT2 Inhibitor-Induced Acute eGFR Dip in CKD Stages 3–4: A Three-Stage Urinary Proteomics Study

**DOI:** 10.3390/life16060865

**Published:** 2026-05-22

**Authors:** Zhiyu Duan, Youhe Gao, Mengjie Huang, Yanjun Liang, Jing Hao, Jie Wang, Guangyan Cai

**Affiliations:** 1 Department of Nephrology, First Medical Center of Chinese PLA General Hospital, National Key Laboratory of Kidney Diseases, National Clinical Research Center for Kidney Diseases, Beijing Key Laboratory of Kidney Diseases Research, Beijing 100853, China; loveduanzhiyu@613.com (Z.D.); huangmengjie301@163.com (M.H.); m17526863652@163.com (Y.L.); 13051669932@163.com (J.H.); 18392931291@163.com (J.W.); 2 Gene Engineering Drug and Biotechnology Beijing Key Laboratory, Department of Biochemistry and Molecular Biology, Beijing Normal University, Beijing 100875, China; gaoyouhe@bnu.edu.cn

**Keywords:** SGLT2 inhibitor, acute eGFR decline, chronic kidney disease, urinary proteomics, prediction model

## Abstract

**Introduction:** SGLT2 inhibitors reduce renal composite endpoints and proteinuria, yet RCTs uniformly show an acute eGFR dip within 2 weeks to 2 months after initiation. However, demographic and clinical predictors of an acute eGFR dip demonstrate considerable heterogeneity across studies. This study aims to identify urinary protein biomarkers of this early eGFR dip and integrate them with routine variables to build a clinically actionable prediction model. **Methods and analysis:** This three-stage proteomics study includes retrospective discovery, prospective internal validation, and external validation cohorts (total *n* ≈ 600–700). DIA mass spectrometry will screen for urinary proteins associated with ≥10% eGFR decline at 1 month post-SGLT2i initiation in CKD stages 3–4. Top candidates (FDR < 10%, FC > 1.5, ion intensity > 1 × 10^4^, unique gene families) will be validated by ELISA. A LASSO-logistic regression model will integrate the top three proteins with seven routinely available clinical variables: age, BMI, diabetes status, heart failure, systolic blood pressure, baseline eGFR, and diuretic use. Model performance will be assessed using the C-statistic, NRI, IDI, and calibration metrics. Adaptive stopping rules are pre-specified. **Ethics and dissemination:** Approved by the Ethics Review Committee at Chinese PLA General Hospital (S2025-859-02, 2025KY126-KS002), all participants will provide written informed consent prior to enrollment, and the study will adhere to the Declaration of Helsinki. Data will be pseudonymized and stored securely according to institutional regulations. Findings will be published in peer-reviewed journals and presented at international nephrology conferences. **Trial Registration:** Registered Report Identifier: ChiCTR2600119772. Date of registration: 3 March 2026.

## 1. Background

Chronic kidney disease (CKD) affects over 800 million individuals globally and remains a leading cause of mortality with rising disease burden [1,2,3,4]. Renin–angiotensin system inhibitors (RASI) established the foundation of CKD therapy, yet leave substantial residual risk—over 40% of treated patients still experience renal composite endpoints [5,6]. Sodium–glucose cotransporter-2 inhibitors (SGLT2i) reduce renal endpoints and proteinuria when added to RASI, but uniformly cause an acute eGFR decline within 2 weeks to 2 months of initiation [7,8,9]. Post hoc analyses of EMPA-REG OUTCOME and DAPA-CKD report that 28–50% of SGLT2i-treated patients experienced acute estimated glomerular filtration rate (eGFR) decline > 10%, compared with 13–24% in placebo controls [10,11]. Despite partial recovery, eGFR remained persistently below baseline and below comparator groups at 12 weeks to 32 months follow-up [10,11]. In advanced CKD (stage 3b), 80% experience acute declines, with 41% exceeding 10% and eGFR failing to return to baseline by 6 months [12].

Current clinical and demographic predictors demonstrate marked heterogeneity across studies and insufficient predictive power [10,11,13]. This study aims to identify urinary protein biomarkers for predicting acute eGFR decline and integrate them with routine variables to develop a clinically actionable prediction model for risk stratification prior to SGLT2i initiation in CKD stages 3–4.

## 2. Materials and Methods

### 2.1. Study Aims and Hypothesis

The primary aim of this study is to develop a predictive model for acute eGFR decline (≥10% at 1 month) in patients with CKD stages 3–4 initiating SGLT2 inhibitor therapy. The model integrates demographic, clinical, and urinary protein biomarker data. Secondary aims include comparing discrimination and calibration between clinical-only and composite models using the C-statistic, net reclassification improvement (NRI), and integrated discrimination improvement (IDI). We evaluate the ability of the final model to stratify patients into low-, intermediate- and high-risk groups for acute eGFR decline, and to compare the actual event rate across strata. We hypothesize that the multimarker model will enable early, non-invasive prediction of acute eGFR decline in CKD stages 3–4 patients initiating SGLT2 inhibitors, allowing preemptive identification of high-risk patients to guide treatment adjustments.

### 2.2. Biomarker Discovery Strategy

The discovery phase employs a systematic, pre-specified analytical pipeline to identify robust urinary protein biomarkers. Briefly, DIA-MS raw files will be processed with DIA-NN v1.8.1 in library-free mode against the UniProt Swiss-Prot human reference proteome (UP000005640), restricted to reviewed canonical entries (approximately 21,000 sequences). A project-specific spectral library will be generated using deep-learning-based neural-network prediction with match-between-runs enabled to augment identification. Protein and precursor-level false-discovery rates will be set to <1%.

Candidate biomarker selection follows a four-tier filtering strategy: (i) magnitude of change—fold change (FC) > 1.5 or <0.67, balancing sensitivity for biomarker discovery with specificity for clinical relevance; (ii) statistical significance—FDR < 5% (Benjamini–Hochberg adjusted *p*-value < 0.05) to control false discovery in high-dimensional data; (iii) technical reliability—mean ion intensity > 1 × 10^4^ ensuring quantification precision and inter-batch consistency; and (iv) biological diversity—retention of top 3 proteins from distinct gene families (defined by UniProt gene symbol) with <50% peptide sequence overlap, capturing complementary pathophysiological mechanisms of SGLT2i-induced hemodynamic changes.

This tiered approach prioritizes proteins that are technically robust, statistically significant, biologically diverse, and amenable to clinical translation via ELISA validation.

### 2.3. Study Design and Settings

**Figure 1:**
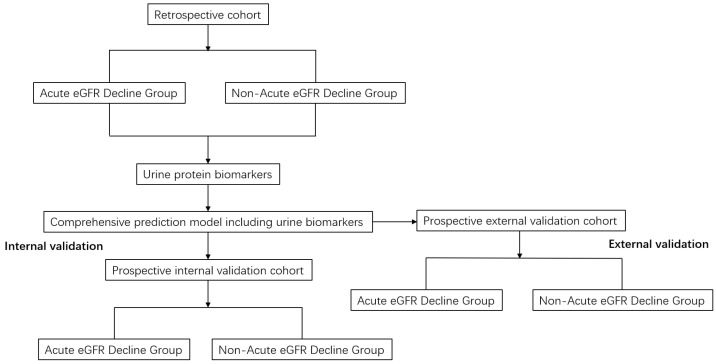
Study design.

This study is a three-stage biomarker research [14]. Firstly, DIA mass spectrometry will be used for preliminary screening of urine protein biomarkers in a retrospective cohort (First Medical Center of the People’s Liberation Army General Hospital). From the ranked list, we will retain the top 3 proteins that simultaneously achieve: (i) FDR  <  5%, fold-change  >  1.5, mean ion intensity  >  1 × 10^4^; (ii) only one representative per protein family (defined by UniProt gene symbol); (iii) no peptide sequence overlap >50% between retained candidates. If fewer than 3 unique families meet the criteria, the discovery set will be expanded until the target is reached. Subsequently, a prospective internal validation cohort will be established at the same center during a different period, using ELISA to validate candidate biomarkers and develop a comprehensive prediction model incorporating demographic, clinical, and protein biomarker data. Finally, a prospective external validation cohort will be recruited at a distinct center (the Fourth Medical Center of the People’s Liberation Army General Hospital) to externally validate the urinary protein biomarkers and the comprehensive prediction model. We will recruit patients with CKD stages 3–4 who are admitted for any reason and who will initiate SGLT2 inhibitor therapy during their hospitalization. Follow-up data at 1 and 12 months post-SGLT2 inhibitor initiation will be collected via clinic visits, local hospital review, or telephone survey, capturing serum creatinine, potassium, urine albumin-to-creatinine ratio, 24 h urine protein, and adverse drug reactions. The study design flow chart is illustrated in Figure 1. This study will follow the Strengthening the Reporting of Observational Studies in Epidemiology guidelines for reporting cohort studies [15]. The study is registered with https://www.chictr.org.cn (ChiCTR2600119772, accessed on 3 March 2026).

### 2.4. Variable Selection and Model Development

We followed a three-step, pre-specified strategy to avoid overfitting and selective reporting. We first conducted a systematic search (PubMed and Embase, 2008–2025) using the keywords “SGLT2 inhibitor”, “acute eGFR decline” and “predictor”. Variables reported in ≥3 independent studies or in one meta-analysis were included in the initial pool. We retained only variables that (i) are routinely available on the day of hospital admission, (ii) have <20% missing values or can be reliably imputed, and (iii) are not deterministically related to the outcome (e.g., eGFR decline). Candidate variables were entered into a LASSO-logistic regression model with 10-fold cross-validation. The tuning parameter (λ) yielding the minimum cross-validated deviance (λ.min) was used to select predictors. Continuous variables were standardized prior to LASSO; highly collinear pairs (|r| > 0.7) were reduced to the clinically simpler one. Between “diabetes status” [16,17,18,19] and “diabetes duration” [20,21,22], we retained the former because it is binary, routinely available, and less collinear with age; duration was thus excluded. References [11,16,17] are post hoc analyses of the same parent trial (DAPA-CKD); they are treated as one source for variable counting. Finally, we included seven routinely available baseline predictors—age [10,13,17,19,20,21,22], BMI [10,13,21,22], diabetes status [16,17,18,19], heart failure [16,21,23], systolic blood pressure [10,16,20,21], eGFR [11,17,18,19,20,21,22] and baseline diuretic use [10,11,13,16,21,22]—in the prediction model. The final set of predictors is shown in Table 1. Missing values (<20%) were imputed using multivariate imputation by chained equations (m = 20, iterations = 50) with predictive mean matching, including age, sex, eGFR, HbA1c, and SBP as predictors. Urine Protein X/Y/Z concentrations were log-transformed (natural log) to achieve approximate normality and to stabilize variance; results are presented as log pg/mL, with a one-unit increase corresponding to an approximately 2.72-fold rise in raw concentration.

### 2.5. Trial Population

The target population of the retrospective cohort is CKD stage 3–4 patients aged 18 to 80 years who were hospitalized in the Nephrology Department of the First Medical Center of the Chinese People’s Liberation Army General Hospital between 1 January 2018 and 1 July 2025. The target population of the prospective internal validation cohort is CKD stage 3–4 patients aged 18 to 80 years who will be hospitalized in the Nephrology Department of the First Medical Center of the Chinese People’s Liberation Army General Hospital between 1 April 2026 and 1 October 2027. The target population of the prospective external validation cohort is CKD stage 3–4 patients aged 18 to 80 years who will be hospitalized in the Nephrology Department of the Fourth Medical Center of the Chinese People’s Liberation Army General Hospital between 1 April 2026 and 1 October 2027. All patients must have a baseline eGFR between 20 and 60 mL/min/1.73 m^2^, plan to initiate SGLT2 inhibitors, and provide written informed consent prior to enrollment. Patients must meet the inclusion and exclusion criteria.

### 2.6. Inclusion and Exclusion Criteria

**Inclusion Criteria**

(1) Adult patients aged 18–80.
(2) Baseline eGFR between 20 and 60 mL/min. 1.73 m^2^.
(3) Urinary albumin creatinine ratio greater than 100 mg/g or 24 h urinary protein quantification greater than 0.3 g/d.

**Exclusion criteria**

(1) Type 1 diabetes mellitus.
(2) Polycystic kidney.
(3) Lupus nephritis.
(4) Antineutrophil cytoplasmic antibody-associated vasculitis.
(5) Anti-glomerular basement membrane disease.
(6) Previously underwent dialysis or kidney transplantation.
(7) Combined urinary tract infections (including pyelonephritis, cystitis, prostatitis, urethritis, etc.).
(8) Pregnant or lactating patients.
(9) Failure to provide a signed informed consent form.
(10) Current SGLT2 inhibitor treatment.
(11) Acute kidney injury within one month prior to enrollment.

**Primary endpoint**

After one month of medication, the acute reduction in eGFR is ≥ 10%.

**Secondary endpoints**

- Adverse reactions to important related drugs include ketoacidosis, urinary tract infections, genital infections, hypoglycemia, necrotizing fasciitis of the perineum, etc.
- Changes in urinary albumin creatinine ratio or 24 h urinary protein quantification after 1 month.
- Absolute value of eGFR change and eGFR slope after 12 months.
- Incidence of hyperkalemia.

**Endpoint definitions**

**Baseline eGFR.** The baseline eGFR is defined as the most recent serum creatinine measurement obtained prior to the first dose of SGLT2 inhibitor (strictly pre-dose, drug-naïve state) within 7 days before dosing, after exclusion of acute kidney injury (AKI). Measurements obtained at any time after the first dose are strictly excluded. If multiple pre-dose measurements are available within this 7-day window, the arithmetic mean will be used. If no pre-dose measurement is available within 7 days, the closest pre-dose measurement within 14 days will be used in a sensitivity analysis only.

**One-month eGFR.** The 1-month eGFR value is defined as the first serum creatinine measurement obtained between day 28 and day 35 after SGLT2 inhibitor initiation. If multiple measurements are available in this window, the value closest to day 30 will be used for the primary analysis.

**Acute eGFR dip.** Calculated as: [(1-month eGFR − Baseline eGFR)/Baseline eGFR] × 100%. A dip ≥ 10% constitutes the primary endpoint.

### 2.7. Patient Identification

Patients will be identified using electronic medical record systems in two hospitals. Details are provided in the Supplementary Material.

### 2.8. Data Collection

All baseline data for patients included will be obtained directly from the electronic medical record system during their hospitalization or after discharge. The baseline data recorded includes age, gender, BMI, medical history (including diabetes, hypertension, stroke, coronary heart disease, heart failure, etc.), systolic and diastolic blood pressure recorded on the day of admission, fasting blood glucose, glycosylated hemoglobin, serum albumin, pre-dose serum creatinine within 7 days, serum cystatin C, eGFR, serum potassium, hematuria (urine red blood cells > 5/high power field of vision or >28/µL), urinary albumin creatinine ratio and (or) 24 h urine protein quantification. Concomitant medications will be recorded, including angiotensin-converting enzyme inhibitors (ACEIs) or angiotensin receptor blockers (ARBs), diuretics, finerenone, glucagon-like peptide-1 receptor agonists, statins, insulin, and glucocorticoids. eGFR will be estimated using the 2021 CKD-EPI creatinine equation without race [24]. The equation is implemented as follows: for females, eGFR = 142 × min (Scr/0.7, 1)^(–0.241) × max (Scr/0.7, 1)^(–1.200) × 0.9938^Age × 1.012; for males, eGFR = 142 × min (Scr/0.9, 1)^(–0.302) × max (Scr/0.9, 1)^(–1.200) × 0.9938^Age, where Scr is serum creatinine in mg/dL and Age is in years. Serum creatinine will be measured using IDMS-traceable enzymatic or compensated Jaffe methods and reported in μmol/L; for eGFR calculation, Scr will be converted to mg/dL by dividing by 88.4. Both centers use IDMS-traceable calibrators and participate in national external quality assessment. Follow-up data will be collected at 1 and 12 months after SGLT2 inhibitor initiation through readmission or telephone follow-up. These data include serum creatinine, serum potassium, urinary albumin creatinine ratio and/or 24 h urine protein quantification, adverse drug reactions, and information on whether dialysis or kidney transplantation has been performed.

### 2.9. Safety Events

Important adverse reactions related to SGLT2 inhibitors, such as ketoacidosis, urinary tract infections, genital infections, hypoglycemia, and necrotizing fasciitis of the perineum, will be monitored and recorded.

### 2.10. Sample Size Calculation

The study employs a three-stage pipeline. Power calculations were anchored to the final 10-variable prediction model (3 proteins + 7 clinical variables). Assuming 15 events per variable and 50% event rate, 150 events (≈300 patients) are required for Stage 2; allowing 10% attrition, a maximum of 330 patients. Stage 3 requires 100 events for C-statistic estimation (95% CI half-width ≤ 0.10), yielding a maximum of 220 patients.

To minimize patient burden and optimize resource allocation, adaptive stopping rules are pre-specified for each stage (see Supplementary Methods). Stage 1 (Discovery): After initial analysis of 50 patients (≈25 events), discovery will terminate if ≥3 proteins from distinct gene families simultaneously achieve FDR  <  5%, fold-change  >  1.5, and mean ion intensity  >  1 × 10^4^; otherwise, recruitment continues in increments of 30 up to a maximum of 150 patients. Stage 2 (Internal validation): An interim blinded evaluation will be performed after 60% of planned recruitment (≈198 patients). If the optimism-corrected C-statistic reaches ≥ 0.80 with a lower 95% confidence limit of ≥0.75 and the calibration slope falls within 0.9–1.1, further enrollment will be halted for futility. Stage 3 (external validation): Early stopping is permitted after 100 patients if the C-statistic estimation achieves the target precision (95% CI half-width ≤ 0.10), rather than continuing to the maximum of 220 patients. Recruitment will occur between 1 January 2026 and 1 January 2028. Total enrollment is anticipated to be 600–700 individuals.

### 2.11. Urine Sample Collection and Mass Spectrometry Analysis

Whole stream early morning urine specimens collected from included patients will be processed within 4 h after collection at 4 °C. To ensure a uniform and clinically applicable prediction tool, all urine specimens will be collected within 24 h before the first dose of SGLT2 inhibitor (drug-naïve state). No SGLT2 inhibitor exposure is required at any stage; other background therapies (ACEI, ARB, diuretics, etc.) remain unchanged and are recorded as covariates. This single-time-point design avoids acute pharmacodynamic interference and maximizes external validity. Each urine sample will be centrifuged at 3000 rpm for 10 min. The urine supernatant will be stored at −80 °C until used. Samples will be thawed on ice, and then 1 mM PMSF will be added. The sample will be centrifuged at 4500× *g* for 10 min at 4 °C, and the supernatant will be collected. Protein concentration will be measured using the BCA assay kit. Equal amounts of protein (50 μg per sample, quantified by BCA assay) will be used for tryptic digestion. A total of 8 M urea will be added to 200 μL of the supernatants, then reduced with 10 mM DTT for 45 min at 37 °C and alkylated with 50 mM iodoacetamide for 15 min in a dark room at room temperature. A total of 4× volume of chilled acetone will be added and precipitated at −20 °C for 2 h. After centrifugation, the protein precipitate will be air-dried and resuspended in 200 μL of 25 mM ammonium bicarbonate solution, and digested with sequencing-grade trypsin (Promega) at a 1:50 enzyme-to-substrate ratio (*w*/*w*) overnight at 37 °C. After digestion, peptides will be desalted using a C18 Cartridge, followed by drying with a vacuum concentration meter, concentrated by vacuum centrifugation and redissolved in 0.1% (*v*/*v*) formic acid.

### 2.12. LC-MS/MS Analysis

The samples will be separated using the Vanquish Neo UHPLC nanolift liquid phase system. The mobile phase A will be a 0.1% formic acid aqueous solution, and the phase B is a 0.1% formic acid acetonitrile solution (acetonitrile is 100%). The injection mode is the capture analysis dual column method, where the trap column is PepMap Neo Trap Cartridge (300 μm × 5 mm, 5 μm) and the analysis column is Easy Spark ™ PepMap ™ Neo UHPLC column (150 μm × 15 cm, 2 μm) (Thermo Fisher Scientific, Shanghai, China). The temperature of the analytical column is controlled at 55 °C by an integrated column temperature box, with a sample loading volume of 200 ng, a flow rate of 2.5 μL/min, an effective gradient of 22 min, and a total machine time of 24 min. DIA analysis will be performed using the nano-accelerated Vanquish Neo system (Thermo Fisher Scientific, Waltham, MA, USA) for chromatographic separation, and the samples separated by nano-upgraded high-performance liquid chromatography will be subjected to DIA (data independent) mass spectrometry analysis using an Orbitrap Astral high-resolution mass spectrometer (Thermo Fisher Scientific, Waltham, MA, USA). Detection mode: positive ion, precursor ion scanning range of 380–980 *m*/*z*, primary mass spectrometry resolution of 240,000 at 200 *m*/*z*, Normalized AGC Target of 500%, maximum injection time of 5 ms. MS2 adopts DIA data acquisition mode, with 299 scanning windows set, Isolation Window set to 2 Th, HCD Collision Energy set to 25%, Normalized AGC Target set to 500%, and maximum injection time set to 3 ms.

### 2.13. Protein Identification and Label-Free Quantification

DIA raw files will be processed with DIA-NN v1.8.1 in library-free mode. Search parameters will be pre-specified as follows: carbamidomethylation of cysteine (+57.0215 Da) as a fixed modification; oxidation of methionine (+15.9949 Da) as a variable modification; trypsin/P specificity with a maximum of one missed cleavage; precursor mass tolerance of 10 ppm; and fragment mass tolerance of 10 ppm. A project-specific spectral library will be generated in silico from the UniProt Swiss-Prot reviewed human proteome (approximately 21,000 canonical entries) using deep-learning-based neural-network prediction; match-between-runs (MBR) was enabled to augment identification. The final library will be used to re-analyze all runs. Protein and precursor-level false-discovery rates (FDR) will be set to <1%. Relative protein abundances will be estimated with the built-in DIA-NN LFQ algorithm (an implementation of the MaxLFQ principle), which normalizes samples using median peptide intensity ratios. Only proteins identified with ≥2 unique peptides will be retained for downstream differential-expression analysis.

A pooled quality control (Pooled QC) sample, prepared by mixing equal volumes from all study samples, will be analyzed at the start, middle, and end of each batch to monitor instrument stability. Proteins with <2 unique peptides, detected in <50% of Pooled QC injections, or with Pooled QC CV% > 30% will be excluded. For biomarker candidate selection, we prioritize proteins detected in ≥70% of individual samples to minimize missing data and ensure reliable quantification.

### 2.14. Bioinformatics Analysis

FDR < 5% (Benjamini–Hochberg adjusted *p*-value, q-value < 0.05) and FC > 1.5 or <0.67 will be used as cutoffs for screening differentially expressed proteins. Clustering of multivariate data will be visualized using Heatmap (http://biit.cs.ut.ee/clustvis, accessed on 1 May 2026) [25]. Details are provided in the Supplementary Material.

### 2.15. Validation by ELISA Analysis

ELISA validation will be performed on three proteins from distinct gene families selected based on the largest fold-changes, FDR < 5%, and ion intensity > 1 × 10^4^. Urine samples for ELISA will be processed using the same pre-analytical pipeline as the discovery cohort (centrifugation at 3000 rpm for 10 min, supernatant storage at −80 °C). Prior to assay, total protein concentration will be determined by BCA, and samples will be diluted as needed to ensure readings fall within the linear range of the standard curve. Typically, 50–100 μL of urine supernatant will be loaded per well, with exact volumes determined according to the manufacturer’s instructions of the selected commercial ELISA kits. To avoid batch effects caused by differences in technical platforms, ELISA validation will be conducted separately in the prospective internal validation cohort and the prospective external validation cohort. The retrospective cohort is reserved for discovery by mass spectrometry and will not be re-analyzed by ELISA. Results obtained by ELISA will be compared with the corresponding mass spectrometry intensities using Spearman correlation; a coefficient r > 0.6 will be considered acceptable inter-platform consistency, confirming the cross-platform stability of the candidate biomarkers.

### 2.16. Statistical Analysis

Statistical analysis will be conducted using SPSS 29.0 (IBM Corp., Armonk, NY, USA) or STATA 15.0 (StataCorp LLC, College Station, TX, USA) software. Graphics will be prepared using Prism 10.1.2 software (GraphPad Corporation, San Diego, CA, USA). Normally distributed continuous data are summarized as mean ± standard deviation. Normality testing will be conducted using the Shapiro–Wilk test or Kolmogorov–Smirnov test, depending on the sample size. The homogeneity of variance test, also known as the F-test, will be used to determine whether the variance of a dataset was homogeneous. Comparisons between independent normally distributed continuous datasets with homogeneity of variance will be conducted using the independent sample *t*-test or one-way ANOVA. Non-normally distributed data are summarized using the median and interquartile range, and datasets will be compared using the nonparametric Mann–Whitney U test. Receiver Operating Characteristic (ROC) curves will be used to analyze the predictive performance of selected biomarkers, clinical models, and comprehensive predictive models. Spearman correlation analysis will be used to analyze the correlation between target biomarkers and clinical indicators. For proteomic differential expression, FDR < 5% will be used; for other comparisons, a two-sided *p* < 0.05 will be considered significant.

Additionally, we will develop a foundational predictive model for SGLT2 inhibitor–induced acute eGFR dip using demographic and clinical variables. These differentially expressed proteins will be incorporated to establish a comprehensive prediction model. The fit of the model will be evaluated by calculating the Akaike information criterion (AIC) for the model with and without the inclusion of these urinary protein biomarkers. The C-index, net reclassification improvement index (NRI), and integrated discrimination improvement index (IDI) will also be calculated and used to estimate the increase in the predictive efficacy achieved using urinary protein biomarker expression.

#### Missing Data Handling

**Missingness mechanism and assumption**: Baseline clinical variables with <20% missingness are assumed missing at random (MAR), conditional on observed covariates included in the imputation model. This assumption is justified because baseline data are extracted from the electronic medical record during hospitalization, where missingness primarily reflects incomplete routine testing rather than patient-level selection. However, we acknowledge that 1-month eGFR and biomarker measurements may not be MAR if missingness is driven by unobserved clinical events (e.g., early AKI, SGLT2i discontinuation, dialysis initiation, or hospital transfer). We explicitly evaluate this possibility through sensitivity analyses.

**Missing data reporting:** The proportion of missing data will be reported by variable, cohort (Stage 1/2/3), center (First vs. Fourth Medical Center), and outcome group (dip vs. no-dip) in a dedicated Supplementary table. For the primary endpoint, reasons for missing 1-month eGFR will be classified into pre-specified categories: (i) administrative (lost to follow-up, telephone unreachable); (ii) clinical (early SGLT2i discontinuation, AKI, dialysis initiation, hospitalization elsewhere, death); and (iii) technical (sample processing failure, ELISA below limit of detection).

**Imputation model:** For baseline variables with <20% missingness, multivariate imputation by chained equations (MICE) will be performed with m = 20 imputations and 50 iterations, using predictive mean matching (PMM) for continuous variables and logistic regression for binary variables. The imputation model will include all variables in the final prediction model (age, BMI, diabetes status, heart failure, systolic blood pressure, eGFR, diuretic use, and the three urinary proteins) plus the primary outcome indicator (acute eGFR dip ≥ 10% at 1 month) and auxiliary variables (sex, HbA1c, serum albumin, potassium) to satisfy the MAR assumption. Parameter estimates from the 20 imputations will be pooled using Rubin’s rules.

**Primary outcome:** The primary outcome (1-month eGFR dip) will not be imputed. Patients with missing primary outcome data will be excluded from the primary analysis and addressed via sensitivity analyses.

**Missing not at random sensitivity analyses:** To assess robustness under departures from MAR, three pre-specified sensitivity analyses will be conducted: (i) complete-case analysis restricted to participants with non-missing 1-month eGFR and all baseline predictors; (ii) best/worst-case imputation, where missing 1-month eGFR in the dip group is imputed as no-dip (and vice versa) to bound the effect estimate; (iii) pattern-mixture modeling, assuming that participants with missing 1-month eGFR due to clinical events have a δ-fold higher (or lower) odds of the primary endpoint compared to observed participants, with δ varied over plausible values (δ = 1.5, 2.0, and 3.0) to identify tipping points. For urinary proteins with values below the limit of detection (LOD), a separate sensitivity analysis using left-censored regression (Tobit model) will be performed.

**Handling of competing events:** Patients who initiate dialysis, undergo kidney transplantation, or die within the first month will be identified through electronic record linkage and telephone follow-up. These events preclude measurement of the primary endpoint and will be reported descriptively. In a secondary analysis, inverse probability of censoring weighting (IPCW) will be applied to account for informative censoring.

### 2.17. Model Development and Validation Framework

**Figure 2:**
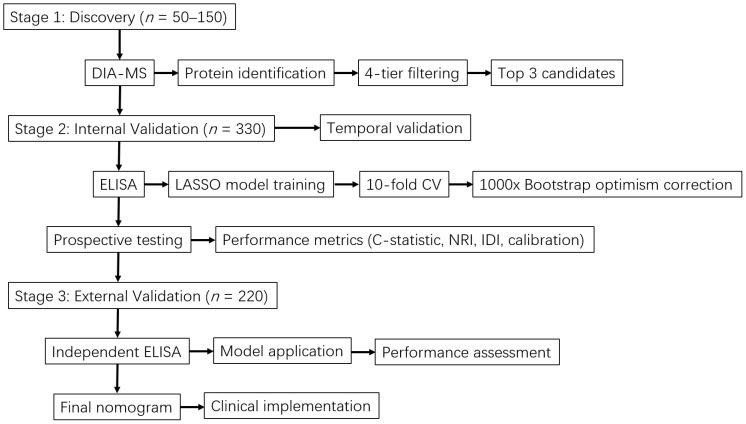
Three-stage biomarker development and model validation workflow. Arrows indicate the sequential flow and linkage between stages, from discovery through internal validation to external validation.

The prediction model (Figure 2) will undergo rigorous three-tier validation to ensure generalizability and clinical utility.

Internal validation: During model training, LASSO-logistic regression with 10-fold cross-validation will be employed. The tuning parameter (λ) will be selected via minimum cross-validated deviance (λ.min). Continuous variables will be standardized prior to LASSO; highly collinear pairs (|r| > 0.7) will be reduced to the clinically simpler variable. Optimism-corrected performance estimates will be derived via 1000 bootstrap resamples, reporting bias-corrected C-statistic and 95% confidence intervals.

Temporal validation: The model will be prospectively applied to the Stage 2 cohort (recruited 2026–2028), distinct from the discovery period, assessing performance stability across time.

External validation: Independent application at the Fourth Medical Center (distinct geographic location and patient population) will evaluate transportability.

Calibration assessment: Calibration slope (target 0.9–1.1) and intercept will assess agreement between predicted and observed probabilities. Decision curve analysis will quantify net benefit across risk thresholds, comparing “treat all,” “treat none,” and model-guided strategies. The final model will be presented as a nomogram for bedside risk calculation.

### 2.18. Sensitivity Analysis

We will conduct pre-specified sensitivity analyses for: (i) alternative endpoint definitions (baseline/1-month windows, ≥15%/≥20% thresholds); (ii) early AKI exclusion; (iii) SGLT2i tolerance stratification; and (iv) concomitant medication effects using a Renal Hemodynamic Load Index. Details are provided in the Supplementary Material.

### 2.19. Data Management and Access

Demographic characteristics, clinical data, and treatment details of enrolled patients will be extracted directly from the electronic medical record system. Details are provided in the Supplementary Material.

Data Sharing and Repository Deposition: Upon completion of the discovery phase, all DIA-MS raw files (.raw), DIA-NN processed outputs (main report and spectral library), and search parameters will be deposited in the PRIDE database (https://www.ebi.ac.uk/pride, accessed on 1 May 2026) or MassIVE (https://massive.ucsd.edu, accessed on 1 May 2026). Sample metadata will be annotated using the Sample and Data Relationship Format (SDRF) to ensure complete traceability between each raw file and the corresponding urine sample. Accession numbers will be provided in the primary results manuscript.

### 2.20. Patient and Public Involvement

Patients and the public were not and will not be involved in the design, conduct, reporting, or dissemination of this research. This is a technical biomarker validation study where the research questions, outcome measures, and analytical methods were defined based on clinical epidemiology principles and prior trial evidence. As this protocol involves laboratory-based biomarker development and statistical model validation rather than behavioral interventions or patient-reported outcome measures, patient involvement in the protocol design was not deemed essential. However, study findings will be disseminated to the patient community through peer-reviewed open access publications and plain-language summaries shared with participating hospital patient education programs.

## 3. Ethics and Dissemination

The study protocol was approved by the local Clinical Research Review Committee and the Institutional Review Board. Approval for the research protocol in this study was obtained from the Ethics Review Committee at the First Medical Center of Chinese PLA General Hospital (S2025-859-02, 27 November 2025) and the Fourth Medical Center of Chinese PLA General Hospital (2025KY126-KS002, 8 December 2025). The research methods adhered to the ethical principles outlined in the Declaration of Helsinki, established by the World Medical Association. Qualified investigators will provide each eligible patient with a full explanation of the study’s objectives, procedures, potential risks, possible adverse reactions, and expected benefits, and will obtain written informed consent before any study-related activities begin. Patients included can withdraw from this study at any time. The research results will be compiled and published in peer-reviewed journals.

### 3.1. Equity, Diversity, and Inclusion

This study aims to enroll a diverse cohort representative of Chinese CKD patients initiating SGLT2 inhibitors. The age range (18–80 years) encompasses both young adults and elderly patients, with planned subgroup analyses by age tertiles (<50, 50–65, >65 years) to evaluate model performance across life stages. Given the demographics of CKD in China, we anticipate balanced sex representation and will perform sex-stratified analyses to ensure predictive biomarkers perform equally in both sexes. The study sites (First and Fourth Medical Centers of Chinese PLA General Hospital) serve diverse populations, including military personnel, retirees, and civilian patients from multiple provinces, enhancing geographic and socioeconomic diversity. However, as a China-based study, generalizability to other ethnic groups (e.g., African, European descent) will be limited; this will be explicitly addressed in the interpretation of findings and highlighted as a priority for external validation in multi-ethnic cohorts.

### 3.2. Clinical Implementation Pathway

The intended clinical use follows a tiered, risk-stratified monitoring protocol designed to reduce SGLT2i discontinuation while ensuring patient safety.

#### 3.2.1. Pre-Treatment Risk Assessment

Prior to SGLT2i initiation, a single urine sample will be collected and analyzed via the validated ELISA panel (turnaround time < 24 h). The multimarker model will generate a predicted probability of acute eGFR decline ≥ 10% at 1 month.

#### 3.2.2. Risk-Stratified Management

As definitive thresholds for this novel prediction model are not established, we propose an exploratory, data-driven approach: risk categories will be defined by the predicted probability distribution in Stage 2 (low: <median; intermediate: median–75th percentile; high: >75th percentile). Final cutoffs will be optimized using X-tile or the Youden index to maximize sensitivity and specificity. Pending validation, the provisional framework includes: (i) low-risk—standard monitoring; (ii) intermediate-risk—enhanced monitoring; (iii) high-risk—nephrology consultation.

#### 3.2.3. Anticipated Clinical Impact

By preemptively identifying high-risk patients, this protocol aims to: (i) reduce anxiety-driven treatment discontinuation; (ii) enable early preventive measures (hydration counseling, diuretic adjustment) in high-risk patients; (iii) optimize resource allocation by reducing unnecessary intensive monitoring in low-risk patients; and (iv) improve long-term cardiorenal outcomes by maintaining SGLT2i therapy in eligible patients.

#### 3.2.4. Implementation Requirements

Successful translation requires: (i) CLIA-waived ELISA assay development (feasibility established by 3-protein panel); (ii) integration with electronic health record systems for automated risk calculation; (iii) clinician education on risk-stratified monitoring protocols; and (iv) patient-facing materials explaining predictive testing and expected eGFR changes.

## 4. Discussion

Glomerular hyperfiltration is one of the mechanisms underlying the occurrence and development of CKD. In the 1980s, observational studies of diabetic nephropathy revealed that early glomerular hyperfiltration predisposed patients to later renal injury; this insight prompted the development and eventual regulatory approval of renin–angiotensin system inhibitors [26]. By lowering intraglomerular pressure, ACEI and ARBs attenuate glomerular hyperfiltration. However, this hemodynamic effect may precipitate an acute, short-term drop in eGFR in CKD patients. In a 1996 RCT of benazepril in CKD patients, serum creatinine rose more steeply in the benazepril arm during the first 2 months; the two groups did not converge until 6 months of follow-up [5]. Post hoc analysis of RENAL [27] showed that losartan-treated patients with an acute eGFR dip began with a higher baseline eGFR than those without a dip, yet ended with a lower mean eGFR after 33 months of follow-up. In addition, patients with acute eGFR decline had a faster eGFR decline slope than those who did not experience acute eGFR decline. A ≥ 30% drop in eGFR following ACEI/ARB initiation is associated with a markedly higher risk of ESRD, myocardial infarction, heart failure, and death in CKD patients; consequently, KDIGO guidelines recommend stopping the drug if this threshold is reached [28]. Even an acute eGFR fall > 10%—well below the 30% discontinuation threshold—significantly raises the risk of ESRD, myocardial infarction, heart failure, and death compared with patients who experience < 10% decline [28]. Thus, an acute eGFR drop > 10% not only heralds worse cardiac and renal outcomes in CKD patients, but also attenuates the protective effects of SGLT2 inhibitors against cardiovascular death, heart-failure hospitalization, and kidney-disease progression [29].

In a real-world cohort of 300,000 patients [28], the incidence of acute eGFR decline greater than 10% after ACEI/ARB drug use was 16.3%, with 1.7% of patients experiencing a decrease of over 30%. In a real-world study of 11,000 individuals initiating SGLT2 inhibitors, 32.4% developed an acute eGFR drop > 10% [13]. A key reason the rate of >10% acute eGFR decline is markedly higher with SGLT2 inhibitors than with ACEI/ARB monotherapy is that most SGLT2 inhibitor users are simultaneously prescribed ACEI/ARBs, and many also receive a mineralocorticoid-receptor antagonist—creating additive intrarenal hemodynamic stress. In DAPA-CKD, >95% of participants were on concomitant ACEI/ARB therapy; despite this background, 49.4% of dapagliflozin-treated patients still had an acute eGFR drop > 10%, and 4.7% experienced a decline > 30% [7]. As RASI, mineralocorticoid-receptor antagonists and SGLT2 inhibitors become increasingly used—often together—the cumulative reduction in glomerular hyperfiltration is expected to drive the incidence of acute eGFR decline even higher. Although acute eGFR dips can develop across any CKD stage, the hazard of a clinically meaningful fall is greatest in advanced disease. For CKD stage 3–4 patients, even a ≥10% swing in eGFR often provokes patient anxiety and prompts patients to lower or stop one of the agents. However, even in CKD stage 5, SGLT2 inhibitors—if continued under close supervision—still lower the risks of dialysis initiation, heart-failure hospitalization, acute myocardial infarction, diabetic ketoacidosis, and AKI [30]. Promptly identifying and managing patients who develop an acute ≥ 10% eGFR decline should help them remain on SGLT2 inhibitor therapy and thereby secure its long-term cardiorenal benefits.

Despite universal awareness of the acute eGFR dip phenomenon, current clinical practice lacks actionable tools for preemptive identification. While post hoc analyses have identified demographic risk factors, the marked heterogeneity across studies limits their individual-level predictive utility. Post hoc analyses of large RCTs and real-world cohorts link acute eGFR decline after SGLT2 inhibitor initiation to factors such as advanced age, baseline CKD, higher HbA1c, and diuretic use; yet the specific predictors and their weights vary markedly across studies [10,11]. A post hoc analysis of EMPA-REG OUTCOME identified diuretic use, higher KDIGO risk category, baseline renal dysfunction, and an acute eGFR drop > 10% as independent predictors of this early dip [11]. Post hoc analysis of DAPA-CKD showed that dapagliflozin-treated patients who sustained an acute eGFR drop > 10% were more often elderly, had higher BMI and systolic blood pressure, lower hemoglobin, a history of smoking, or were on diuretics [10]. Yet the post hoc analysis of DAPA-HF found that only advanced age and diabetes were significant predictors of an acute eGFR drop > 10% in the dapagliflozin arm [19]. A Taiwanese multicenter real-world study further showed that diuretic or insulin use, prior stroke, advanced age, female sex, high HbA1c, and BMI < 25 kg/m^2^ independently predicted an acute eGFR fall > 30%, whereas statin use was protective [13]. Thus, predictors of acute eGFR drop differ across SGLT2 inhibitors, populations, and even between trials of the same agent; relying on demographic and routine clinical variables alone is insufficient to foretell this early dip. Consequently, clinicians face a binary dilemma: either withhold SGLT2i from all advanced CKD patients (denying them long-term cardiorenal benefits) or prescribe universally and manage reactive anxiety when dips occur. Our model addresses this therapeutic paralysis by enabling personalized risk stratification prior to drug initiation. This model aligns with the KDIGO 2024 strong recommendation to initiate SGLT2i in patients with type 2 diabetes and CKD stages G3–G4 (eGFR ≥ 20 mL/min/1.73 m^2^) by providing a practical implementation tool [31]. Rather than replacing clinical judgment, the multimarker score quantifies the risk-benefit ratio to support shared decision-making, particularly in the ‘gray zone’ of eGFR 30–45 mL/min/1.73 m^2^, where physician prescribing hesitancy is highest.

CKD273 peptide classifier was first discovered in 2010 as a set of biomarkers for CKD diagnosis [32]. Subsequent studies have confirmed that it is still a good predictive tool for CKD progression [33,34]. In a double-blind, placebo-controlled, crossover trial of 40 type 2 diabetic patients with UACR ≥ 30 mg/g, dapagliflozin significantly lowered the CKD273 score and shifted a greater proportion of participants from the high-risk to the low-risk proteomic pattern compared with placebo [35]. Dapagliflozin treatment mainly affects 36 urinary peptide fragments from 19 proteins, which are related to biological processes such as inflammation, wound healing, and renal fibrosis [36]. However, CKD273 was only found to be positively correlated with baseline UACR, and the changes in CKD273 after treatment with dapagliflozin were not related to changes in eGFR. For type 1 diabetes patients with normal renal function, though the expression of 107 urinary peptide segments was significantly changed after treatment with empagliflozin, and most of the changes were in the opposite direction to those in CKD patients, that is, toward the “healthy” direction, there was no significant impact on the CKD273 score [37]. The possible reason is speculated to be that the research subjects themselves have normal kidney function, and their CKD273 scores are very low. CKD273 scores are not related to the changes in eGFR after SGLT2i treatment. To date, SGLT2 inhibitor proteomic studies have focused on elucidating drug mechanisms [38,39]; none have yet used protein signatures to predict either long-term efficacy or the acute eGFR dip. In contrast, accumulating evidence shows that urinary-proteome profiles reliably forecast both the short-term [40] and long-term [41,42] therapeutic response to RAS inhibitors in CKD patients.

Real-world data reveal that SGLT2 inhibitor prescribing falls sharply as CKD stage advances [43]. In a Singaporean cohort of 4446 type 2 diabetic patients, 47.2% of those with baseline eGFR ≥ 60 mL/min/1.73 m^2^ were prescribed an SGLT2 inhibitor, whereas the prescription rate fell to only 19.6% among patients with eGFR < 60 mL/min/1.73 m^2^ [43]. A UK real-world study reported that only 1.7% of patients with eGFR < 60 mL/min/1.73 m^2^ were newly started on an SGLT2 inhibitor [44]. Dehydration and short-term acute decline in renal function may be the reasons for low prescribed SGLT2 inhibitors in advanced CKD patients [45]. As CKD stage advances, the proportion of patients experiencing an acute > 10% eGFR decline after SGLT2 inhibitor initiation rises markedly [11]. Post hoc analysis of DAPA-CKD showed that, with more than 90% of participants having eGFR < 60 mL/min/1.73 m^2^, the dapagliflozin arm exhibited a 49.4% incidence of acute eGFR decline > 10% [10]. Because acute eGFR dips occur in roughly half of CKD stages 3–4 patients, prescribers often withhold SGLT2 inhibitors from this group; an early, reliable prediction tool would reassure clinicians and materially increase SGLT2 inhibitor uptake in this population.

Several limitations should be considered when interpreting the findings of this study. First, the study is conducted exclusively in the Chinese Han populations at two hospitals. While this enhances internal validity, generalizability to other ethnic groups (e.g., African, European, or South Asian descent) may be limited due to potential differences in CKD progression patterns, SGLT2i metabolism, and baseline proteinuria levels. External validation in multi-ethnic cohorts is a priority for future research, as explicitly acknowledged in our equity and diversity statement. Second, although the 10-variable multimarker model is parsimonious by design, the inclusion of only three proteins from distinct gene families represents a trade-off between clinical feasibility and biological comprehensiveness. This approach may exclude proteins with moderate but genuine predictive value that could enhance model discrimination in specific subgroups. The adaptive stopping rules, while ethically necessary, may also result in a discovery cohort at the lower bound of proteomic depth. Third, despite rigorous variable selection via LASSO regression, residual confounding from unmeasured factors (e.g., subtle volume status, sodium intake, or genetic variants in SGLT2 transporters) cannot be excluded. The Renal Hemodynamic Load Index in sensitivity analyses captures medication effects but not dietary or environmental determinants of glomerular hemodynamics.

This study possesses several methodological and clinical strengths. First, to our knowledge, this represents the first prospective biomarker study specifically designed to predict SGLT2i-induced acute eGFR decline in CKD stages 3–4. Unlike post hoc analyses of RCTs constrained by restrictive eligibility criteria, our protocol recruits real-world hospitalized patients with complex comorbidities, enhancing external validity for the target clinical population. Second, the three-tier validation framework—comprising discovery, internal temporal validation, and external geographic validation—mitigates overfitting and ensures model generalizability across different clinical settings and time periods. The pre-specified adaptive stopping rules optimize resource efficiency while maintaining statistical rigor, representing an ethical and scientific advancement over traditional fixed-sample designs.

In short, this study will be the first to mine urinary-proteome data for non-invasive protein biomarkers that foreshadow an acute > 10% eGFR fall after SGLT2 inhibitor initiation in CKD stages 3–4 patients, and will fuse these biomarkers with demographic and clinical variables to build a more accurate, clinically actionable prediction model. If validated, the 3-protein signature will be amenable to development as a rapid ELISA-based diagnostic assay for prospective risk stratification prior to SGLT2 inhibitor initiation.

## Figures and Tables

**Table 1:** Final predictor set entered the prediction model.

Variables	Type	Unit	Data Missing Handling	Literature Source
Age	Continuous	Years	-	[11,13,17,19,20,21,22]
BMI	Continuous	kg/m^2^	<20% missing, MICE	[11,13,21,22]
Diabetes	Binary	Yes/No	-	[16,17,18,19]
Heart failure	Binary	Yes/No	-	[16,21,23]
Systolic blood pressure	Continuous	mmHg	<20% missing, MICE	[11,16,20,21]
eGFR	Continuous	ml/min/1.73 m^2^	-	[10,17,18,19,20,21,22]
Use diuretics at baseline	Binary	Yes/No	-	[10,11,13,16,21,22]
Protein X (ELISA)	Continuous	log pg/mL	MICE	
Protein Y (ELISA)	Continuous	log pg/mL	MICE	
Protein Z (ELISA)	Continuous	log pg/mL	MICE	

BMI, body mass index; MICE, multivariate imputation by chained equations; eGFR, estimated glomerular filtration rate; variable selection and model development.

## Data Availability

The study protocol does not contain primary experimental data. DIA-MS raw files, processed outputs, and SDRF-annotated sample metadata will be deposited in PRIDE or MassIVE upon completion of the discovery phase, with accession numbers reported in the primary results publication.

## Supplementary Materials

The following supporting information can be downloaded at: https://www.mdpi.com/article/10.3390/life16060865/s1.
