# Supplementary material for: Protocol for Developing and Validating a Multimarker-Clinical Prediction Model of SGLT2 Inhibitor-Induced Acute eGFR Dip in CKD Stages 3–4: A Three-Stage Urinary Proteomics Study
Source: Life (Basel). 2026 May 22;16(6):865. doi: 10.3390/life16060865 (PMC13302278; doi:10.3390/life16060865)
Supplement: Supplementary file 1 [file life-16-00865-s001.zip › life-4284262-supplementary.pdf]

Supplemental Material

Supplementary methods

Patient identification

Patients will be identified using electronic medical record systems in two hospitals. Select eligible patients based on inclusion and exclusion criteria, and collect morning urine samples. The preliminary identification of patients at the First Medical Center of the General Hospital of the Chinese People's Liberation Army will be conducted by nephrologist JW through the electronic medical record system at specific times every Monday, Wednesday, and Friday morning. This process includes a review of newly admitted patients from the past 1 to 2 days to ensure all admitted patients can be screened. Patients at the Fourth Medical Center of the Chinese People's Liberation Army General Hospital will be identified by nephrologist ZYD at the same time point. All patients who have undergone preliminary screening and inclusion, as well as those who meet the inclusion criteria but also meet one or more exclusion criteria, will undergo a secondary review by GYC and XMC to ensure the accuracy of the selection process.

Sample size calculation

The study employs a three-stage biomarker pipeline—discovery, internal validation and external validation—in which only 3 proteins from distinct gene families (largest fold-change, FDR<10%, ion intensity>1×10^4) will be carried forward into the final prediction model; power calculations were therefore anchored to the number of variables in that model rather than to the entire

proteome screened in stage 1.

We will first analyse the blinded DIA dataset of the initial 50 patients (≈25 dip events). If ≥3 proteins from distinct gene families simultaneously achieve FDR < 10%, fold-change > 1.5 and mean ion intensity > $1×10^4$, discovery will be terminated and the remaining banked specimens will be held as reserve. If the criterion is not met, recruitment will continue in increments of 30 patients until either the protein target is reached or 150 patients have been analysed.

The final logistic model will include 10 variables (1 demographic, 6 clinical, and 3 urinary proteins). Assuming 15 events per variable and an expected dip rate of 50%, 150 events (≈300 patients) are required. Allowing for 10% loss to follow-up, a maximum of 330 patients will be enrolled in stage 2.

To estimate the C-statistic with 95% CI half-width ≤0.10, 100 events are needed [1]. Assuming 50% event rate and 10% attrition, a maximum of 220 patients will be recruited in stage 3; early stopping is pre-specified after 100 patients if the above performance criteria are met.

An adaptive stopping rule is pre-specified: if an interim blinded evaluation of the first 60% of stage-2 recruits yields an optimism-corrected C-statistic ≥0.80 with lower 95% CI ≥0.75 and a calibration slope between 0.9 and 1.1, further enrolment will be halted for futility.

Thus, under the adaptive framework the study will enroll at least 50 (expandable to 150) patients in stage 1, no more than 330 in stage 2 and no more than 220 in stage 3, totaling approximately 600–700 individuals, a sample size

sufficient to develop, internally validate and externally confirm a parsimonious 3-protein signature for predicting acute eGFR decline ≥10% one month after SGLT2-inhibitor initiation in CKD stages 3–4.

Bioinformatics analysis

Differential proteins were subjected to enrichment analysis at four levels: Gene ontology (GO) classification, Eucalyptic Orthologous Groups (KOG) functional classification, the Kyoto Encyclopedia of Genes and Genomes (KEGG) pathway, and protein structural domains. The enrichment analysis employs hypergeometric tests to calculate the significance P-value, aiming to identify whether differential proteins exhibit significant enrichment in specific functional categories compared to the background proteins (all identified proteins). Additionally, predictions and analyses regarding subcellular localization and signal peptides are conducted to comprehensively determine the physiological functions implicated by these differential proteins. Protein-protein interaction (PPI) networks were performed with the STRING online database (https://cn.string-db.org/).

Sensitivity analysis

To assess the robustness of the primary findings, we will conduct the following pre-specified sensitivity analyses for the primary endpoint. We will repeat the primary analysis using: (i) alternative baseline eGFR definitions (mean of two most recent measurements within 14 days; single measurement closest to dosing); (ii) alternative 1-month eGFR definitions (mean of all measurements in 28–35 day window; lowest value in 28–42 day window; time-adjusted analysis including day

21–42); (iii) alternative decline thresholds (⩾15% or ⩾20%). To mitigate baseline variability, we will employ multiple eGFR calculation methods. Specifically, baseline eGFR will be calculated using the average of the two most recent serum creatinine measurements obtained prior to treatment initiation. Although the main exclusion criterion already eliminates patients who develop AKI within the first month after enrolment, transient AKI can still occur as early as 7 days after SGLT2-inhibitor initiation and may artifactually lower eGFR. We will therefore perform a sensitivity analysis that further excludes any participant who meets KDIGO AKI criteria during the first 7 days of therapy, to assess whether such early events influence the predictive performance of the protein biomarkers. Additionally, we will perform a subgroup analysis stratified by SGLT2-inhibitor tolerance. The predictive accuracy of urinary protein biomarkers will be compared between the continuous-treatment group (uninterrupted therapy ⩾28 days without dose reduction or discontinuation) and the early-adjustment group (dose reduction or discontinuation within 28 days). We will also conduct sensitivity analysis of concomitant medication effects. Given that baseline diuretic use is already included as a core clinical predictor (Table 1) and that nearly all CKD stages 3–4 patients receive renin-angiotensin system inhibitors (RASI), we will evaluate medication effects through a load-gradient approach rather than simple stratification. A Renal Hemodynamic Load Index will be constructed as a 3-level ordinal variable: Level 0 (neither RASI nor diuretic; expected <5% of cohort), Level 1 (RASI or diuretic alone), and Level 2 (combined RASI + diuretic therapy,

anticipated in 60–70% of patients). For strata with fewer than 50 patients or <15 dip events, descriptive comparison will be performed instead of independent modeling to avoid overfitting. The composite prediction model will be refit including this index as a covariate and its interaction terms with each protein marker (Protein X/Y/Z × Load Index). Interaction effects will be tested at a significance threshold of $P \geqslant 0.10$; values meeting this criterion will be interpreted as absence of statistically significant effect modification by medication load. This approach enables prospective assessment of model robustness across varying background therapy regimens while preserving statistical power and avoiding stratification-induced sample size reduction. All sensitivity analyses were conducted using the same statistical modeling framework as the main analysis (such as LASSO logistic regression, 10-fold cross validation). The results will be reported in the form of odds ratio (OR) and 95% confidence interval, and descriptive comparison of model performance indicators (AUC, sensitivity, specificity, positive predictive value, negative predictive value) will be conducted.

Data management and access

Demographic characteristics, clinical data, and treatment details of enrolled patients will be extracted directly from the electronic medical record system. Demographic characteristics, clinical data, and treatment details of enrolled patients will be extracted directly from the electronic medical record system. All data will be stored in compliance with local regulations and with participants' written informed consent. Privacy-sensitive data include all direct identifiers (e.g.,

name, national ID, full address) and quasi-identifiers (exact dates of birth, death, procedures; rare diagnoses; genomic sequences). These data will be pseudonymised immediately after collection using a centrally generated 12-digit study ID. The linkage file mapping study ID to real identity will be encrypted, and destroyed 15 years after study completion. The encryption key will be available only to coordinating researchers. The study team will be responsible for data management. Hospital independent monitors will perform data validation checks and ensure that all study procedures comply with local regulations and protocol.

REFERENCES

1. Dobbin KK, McShane LM: Sample size methods for evaluation of predictive biomarkers. Stat Med 2022, 41(16):3199-3210.
